# Mesenchymal stem cells-derived exosomal miR-653-5p suppresses laryngeal papilloma progression by inhibiting BZW2

**DOI:** 10.1016/j.clinsp.2022.100129

**Published:** 2022-12-05

**Authors:** Binya Hu, Min Huang, Lihua Tao, Yun Li, Yuting Kuang, Guangliang Liu, Sijun Zhao

**Affiliations:** Department of Otorhinolaryngology, Head and Neck Surgery, Hunan Children's Hospital, China

**Keywords:** Papillomatosis, Laryngeal Diseases, Mesenchymal Stem Cells, MicroRNAs, Exosomes, MSCs, Mesenchymal Stem Cells, LP, Laryngeal Papilloma, BZW2, Basic Leucine Zipper and W2 domains 2, HPV, Human Papilloma Virus, BTG2, BTG anti-proliferation factor 2, PI3K, Phosphatidylinositol 3-Kinase, AKT, AKT serine/Threonine Kinase, CDKL3, Cyclin-Dependent Kinase Like-3, MAPK6, Mitogen-Activated Protein Kinase 6, FBS, Fetal Bovine Serum, EdU, 5-Ethynyl-2’-deoxyuridine, MTT, (4,5)-dimethylthiahiazo (-z-y1)-3,5-di- phenytetrazoliumromide, RIP, RNA Immunoprecipitation, WB, Western Blotting, Qpcr, Quantitative polymerase chain reaction, KDM5C, lysine-specific demethylase 5C, OGFOD1, 2-Oxoglutarate and iron-dependent Oxygenase Domain containing 1, HIPK2, Homeodomain-Interacting Protein Kinase 2

## Abstract

**Objectives:**

Although miR-653-5p has been validated to participate in the progression of multiple types of cancer, the functional role of exosomal miR-653-5p derived from Mesenchymal Stem Cells (MSCs) in Laryngeal Papilloma (LP) has still remained elusive. Hence, this study aimed to investigate the role of MSCs-derived exosomal miR-653-5p in LP.

**Methods:**

LP tissues (*n* = 15) and adjacent normal tissues (*n* = 10) were collected to examine the expression level of miR-653-5p. The expression level of miR-653-5p in LP cells and normal cells was also detected. Then, miR-653-5p was overexpressed or silenced to explore its effects on the proliferation, migration, invasion, and apoptosis of LP cells. Thereafter, the effects of exosomal miR-653-5p derived from MSCs on LP cell progression and the potential regulatory mechanism of miR-653-5p were assessed.

**Results:**

It was revealed that the expression level of miR-653-5p was downregulated in LP tissues and cells. In addition, miR-653-5p suppressed the proliferation, migration, invasion, and apoptosis of LP cells. Exosomes derived from MSCs played a suppressive role in LP development and mediated the transmission of miR-653-5p to LP cells. Further exploration identified Basic leucine Zipper and W2 domains 2 (BZW2) as the target of miR-653-5p. More importantly, the rescue experiments revealed that MSCs-secreted exosomal miR-653-5p efficiently inhibited the aggressive phenotypes of LP cells, which could be significantly reversed by BZW2 overexpression in LP cells.

**Conclusion:**

MSCs-derived exosomal miR-653-5p exerted inhibitory effects on LP progression through targeting BZW2, which provided a novel idea for the therapy of LP.

**Clinical Trial registration number:**

chictr-ior-17011021.

## Highlights

• The downregulation of miR-653-5p is involved in the progression of LP.
• MSCs-derived exosomal miR-653-5p suppressed the malignant behaviors of LP cells.
• The role of MSCs-derived exosomal miR-653-5p in LP relied on BZW2.

## Introduction

Laryngeal Papilloma (LP) is defined as a relatively common benign tumor, which mainly affects children aged under 10 years old,^1^^,^^2^ and it is generally considered to be caused by Human Papilloma Virus (HPV) infection.^3^^,^^4^ Typical symptoms of LP include foreign body sensation in the throat,^5^ cough,^6^ hoarse voice,^7^ and even dyspnea.^2^ Pathologically, LP is classified as an epithelioma, which is composed of stratified squamous epithelium, typically without infiltrated basal tissues.^8^ Laryngoscopy enucleation is widely implemented to alleviate LP.^9^ Despite its benign characteristics, the recurrence rate of LP remains high, and there is a lack of biomarkers for the diagnosis and treatment of LP.

Exosomes are extracellular vesicles with a size of 30‒150 nm derived from a variety of cells,^10^^,^^11^ containing abundant active substances, such as DNA, proteins, lipids, as well as nucleic acids, which mainly consisted of circular RNAs (circRNAs), long noncoding RNAs (lncRNAs), and microRNAs (miRNAs).^12^, ^13^, ^14^ Using bioactive substances, exosomes mediate intercellular communication and signal transmission.^15^

Accumulating evidence demonstrated the remarkable contribution of exosome-derived miRNAs to various biological behaviors of cancer cells, including cell proliferation,^16^ invasion,^17^^,^^18^ differentiation,^19^ and Epithelial-Mesenchymal Transition (EMT).^20^ More specifically, exosome-delivered miR-15a accelerated the proliferation of clear cell renal cell carcinoma cells by targeting the BTG anti-proliferation factor 2 (BTG2)/Phosphatidylinositol 3-Kinase (PI3K)/AKT serine/Threonine Kinase (AKT) axis.^21^ Sun et al. found that exosomal miR-205-5p derived from Bone Marrow Stromal Cells (BMSCs) played an inhibitory role in the tumorigenicity of liver cancer cells via suppressing the expression of Cyclin-Dependent kinase-like 3 (CDKL3).^22^ In addition, Yu et al. suggested that hypoxic tumor-derived exosomal miR-31-5p promoted lung adenocarcinoma metastasis via accelerating the EMT process and activating the MEK/ERK signaling pathway.^23^ In recent years, the role of miR-653-5p in various diseases has been investigated, especially in malignant tumors. For instance, miR-653-5p suppressed the progression of Wilms's tumor.^24^ Besides, miR-653-5p retarded the growth and migration of breast cancer cells by negatively modulating Mitogen-Activated Protein Kinase 6 (MAPK6).^25^ To date, the involvement of Mesenchymal Stem Cells (MSCs)-derived exosomal miR-653-5p in laryngeal papilloma progression has not been reported.

In the present study, the miR-653-5p expression level in LP tissues and cells was measured, and the functional role of MSCs-derived exosomal miR-653-5p in the biological processes of LP cells was assessed. To gain a deep understanding of the LP pathogenesis, this study aimed to shed light on its potential molecular mechanism in LP, which could be advantageous to ameliorate LP.

## Materials and methods

### Tissue collection

LP tissues (*n* = 15) and adjacent normal tissues (*n* = 10) were supplied by Hunan Children's Hospital (Changsha, China), and all experiments were performed based on the guidance of the Declaration of Helsinki. The study protocol was approved by the Ethics Committee of Hunan Children's Hospital (Approval n° chictr-ior-17011021), and all patients signed the informed consent form prior to enrollment.

### Cell culture and transfection

MSCs were obtained from Saliai Stem Cell Science and Technology (Guangzhou, China), and the medium supplemented with 10% Fetal Bovine Serum (FBS) was employed for the cultivation of MSCs. After isolation of LP and normal cells from LP and adjacent normal tissues, cells were cultured at 37°C in the medium containing 10% FBS, 100 μg/mL streptomycin, and 100 IU/mL penicillin.

For cell transfection, mimics and inhibitors of miR-653-5p and pcDNA-BZW2 plasmids were produced by GeneChem Co., Ltd. (Shanghai, China).

### Isolation and identification of exosomes

Total exosome isolation reagent (#4478359; Thermo Fisher Scientific, Waltham, MA, USA) was utilized to isolate exosomes from MSCs as per the protocol. Next, transmission electron microscopy (TEM; Philips CM120 BioTwin, FEI Inc., New York, NY, USA) was applied for the characterization of exosomes as previously reported.^26^

### Cell proliferation assay

For the assessment of MSC proliferation, a BeyoClick™ 5-Ethynyl-2’-Deoxyuridine (EdU) Cell Proliferation kit (Beyotime Institute of Biotechnology, Shanghai, China) was utilized based on the manual. Nuclear staining was performed using 4′,6-Diamidino-2-Phenylindole (DAPI), and a fluorescence microscope (Olympus, Tokyo, Japan) was used for the measurement of EdU-incorporated cells.

For 3-(4,5)-dimethylthiahiazo (-z-y1)-3,5-di-phenytetrazoliumromide (MTT) assay, MSCs were cultivated in 96-well plates after 48h of transfection. 5-days later, 10 µL of MTT solution was added to each well. A microplate reader was utilized to detect the absorbance at 490 nm.

### Cell migration and invasion assays

A wound healing assay was carried out to measure the migration of LP cells. LP cells were seeded into a 6-well plate and cultivated until a 70%‒80% confluence was reached. The scratch was created using a 200 µL pipette tip. Then, the wound was monitored and recorded under a microscope after 0 and 48h of incubation.

For transwell invasion assay, transwell chamber (8-μm pore size; Corning Inc., Corning, NY, USA) and matrigel (BD Biosciences, Franklin Lakes, NJ, USA) were utilized. LP cells resuspended in 200 μL of a serum-free medium were placed in the upper chamber. Meanwhile, the lower chamber was supplemented with 600 μL of complete medium and 10% FBS. After incubation for 48h, invaded cells were subjected to fixation in methanol, dyed with crystal violet, and counted under a microscope.

### Dual-luciferase reporter assay

For correlation confirmation, basic leucine zipper and W2 domains 2 (BZW2)-WT and BZW2-MUT were either co-transfected into LP cells with miR-653-5p inhibitor/NC inhibitor or with exosomes from MSCs exposed to miR-653-5p mimic/NC mimic. Then, the measurement of the luciferase activity of BZW2 was conducted using a Dual-Luciferase Reporter Assay system (Promega, Madison, WI, USA).

### RNA immunoprecipitation (RIP) assay

A Magna RNA-Binding Protein Immunoprecipitation kit (Millipore, Billerica, MA, USA) was applied to conduct the RIP assay. In brief, cells were lysed in RIPA lysis buffer, and the collected cell lysates were mixed with anti-AGO2 (ab222699; Abcam, Cambridge, UK) or IgG (Cat. #PP64B; Millipore) and magnetic beads. After digesting proteins by adding proteinase K, the quantitative Polymerase Chain Reaction (qPCR) was performed to assess the enrichment of BZW2.

### Western Blotting (WB)

In short, the lysis buffer and Phenylmethylsulfonyl Fluoride (PMSF) were used to extract the protein. After that, 10% Sodium Dodecyl-Sulfate Polyacrylamide Gel Electrophoresis (SDS-PAGE) was used to separate the collected proteins. After transferring proteins to Polyvinylidene Difluoride (PVDF) membranes, the membranes were incubated overnight at 4°C with the following primary antibodies: CD9 (ab92726, 1:2000), CD63 (ab134045, 1:1000), CD81 (ab79559, 2 µg/mL), Bax (ab32503, 1:2000), Bcl-2 (ab32124, 1:1000), caspase-3 (ab32351, 1:5000), cleaved caspase-3 (ab32042, 1:500), caspase-9 (ab32539, 1:1000), cleaved caspase-9 (ab2324, 1 µg/mL), MMP-1 (ab134184, 1:1000), MMP-9 (ab76003, 1:5000), BZW2 (ab254772, 0.4 µg/mL), and Glyceraldehyde-3-Phosphate Dehydrogenase (GAPDH) (ab9485, 1:2500). Following incubation with anti-mouse/rabbit IgG antibody conjugated to horseradish peroxidase (HRP) for 1h, enhanced chemiluminescence reagent (Thermo Fisher Scientific) was used for visualization. GAPDH was utilized as the loading reference.

### qPCR assay

For RNA quantification, TRIzol reagent (Thermo Fisher Scientific) was used for the extraction of total RNA from tissues and cells. Using the Reverse Transcription kit (Takara, Dalian, China), RNA samples were reversely transcribed into cDNA. Then, qPCR was carried out on ABI 7500 PCR system (Applied Biosystems, Waltham, CA, USA) utilizing Power SYBR™ Green PCR master mix (Thermo Fisher Scientific). The expression levels of miR-653-5p and BZW2 were normalized to those of U6 and GAPDH, respectively. The primer sequences used for qPCR are listed in Supplementary Table 1.

### Statistical analysis

All data were processed and analyzed by GraphPad Prism 7 software (GraphPad Software Inc., San Diego, CA, USA), and were shown as mean ± Standard Error of the Mean (SEM). ImageJ software (National Institutes of Health, Bethesda, MD, USA) was applied for making a comparison between groups. The correlation between miR-653-5p and BZW2 was analyzed by Spearman's correlation coefficient; *p* < 0.05 was considered statistically significant.

## Results

### Overexpression of miR-653-5p suppressed proliferation, migration, and invasion of LP cells

**Fig. 1:**
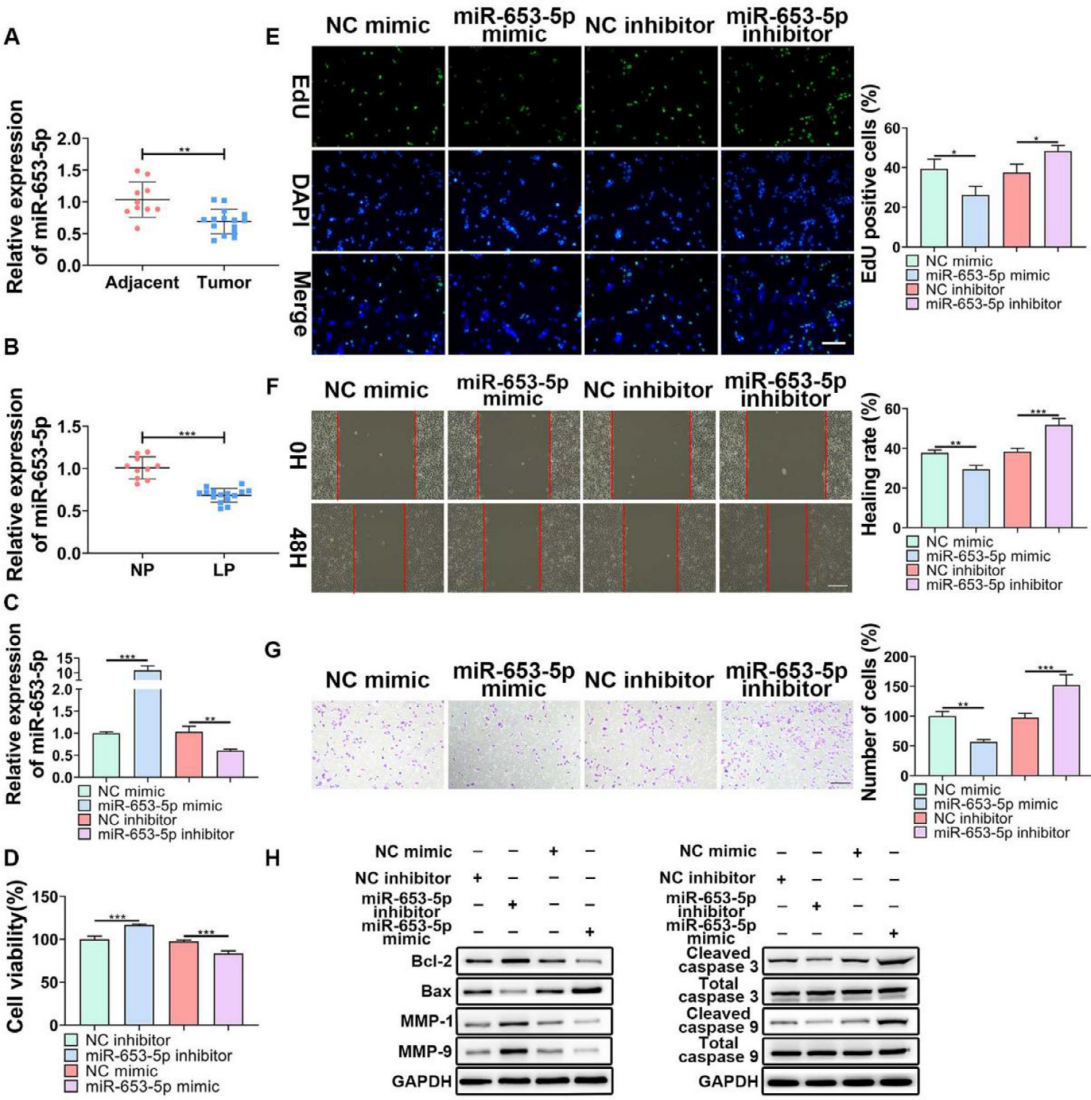
**The effects of miR-653-3p on proliferation, migration, and invasion of LP cells.** (A) qPCR analysis of miR-653-5p expression in LP tissues and adjacent normal tissues (B) qPCR analysis of miR-653-5p expression in LP cells and normal pharyngeal epithelial cells. (C) The transfection efficacy of miR-653-5p overexpression and miR-653-5p knockdown was detected by qPCR. (D, E) MTT (D) and EdU (E) assays were employed to assess LP cell proliferation. (F, G) Wound healing assay (F) and transwell assay (G) were used to assess migration and invasion of LP cells, respectively. (H) WB assay was performed to detect the expression levels of Bax, Bcl2, MMP1, MMP9, caspase-3, cleaved-caspase-3, caspase-9, and cleaved-caspase-9 under miR-653-5p overexpression or knockdown. *p < 0.05, ^⁎⁎^*p* < 0.01, ^⁎⁎⁎^*p* < 0.001.

To determine the influences of miR-653-5p on the biological behaviors of LP cells, its expression level in LP tissues and cells was detected. The results of a q-PCR assay (Fig. 1B) revealed that miR-653-5p was lowly expressed in LP tissues compared with that in adjacent non-tumor tissues (Fig. 1A). In addition, the expression level of miR-653-5p was lower in LP cells than that in normal pharyngeal epithelial cells (Fig. 1B). For further functional investigations, the miR-653-5p-overexpressed or miR-653-5p-silenced LP cells were constructed (Fig. 1C). The results of MTT assay indicated that miR-653-5p overexpression weakened the proliferative ability of LP cells, and knockdown of miR-653-5p provoked the opposite result (Fig. 1D). Moreover, the inhibitory role of miR-653-5p in LP cell proliferation was also validated by the EdU assay (Fig. 1E). It was confirmed by wound healing and transwell assays that overexpression of miR-653-5p suppressed the migration and invasion of LP cells while silencing of miR-653-5p facilitated migratory and invasive capacities of LP cells (Fig. 1 F, G). In addition, miR-653-5p overexpression caused the downregulation of Bcl-2, MMP-1, and MMP-9, as well as the upregulation of pro-apoptotic proteins, including Bax, cleaved caspase-3, and cleaved caspase-9 (Fig. 1H). Depletion of miR-653-5p increased the expression levels of Bcl-2, MMP-1, and MMP-9, whereas reduced the expression levels of Bax, cleaved caspase-3, and cleaved caspase-9 (Fig. 1H). Overall, these findings suggested that miR-653-5p was downregulated in LP cells, and it functioned as a cancer suppressor in LP.

### MSCs-derived exosomes inhibited LP development *in vitro*

**Fig. 2:**
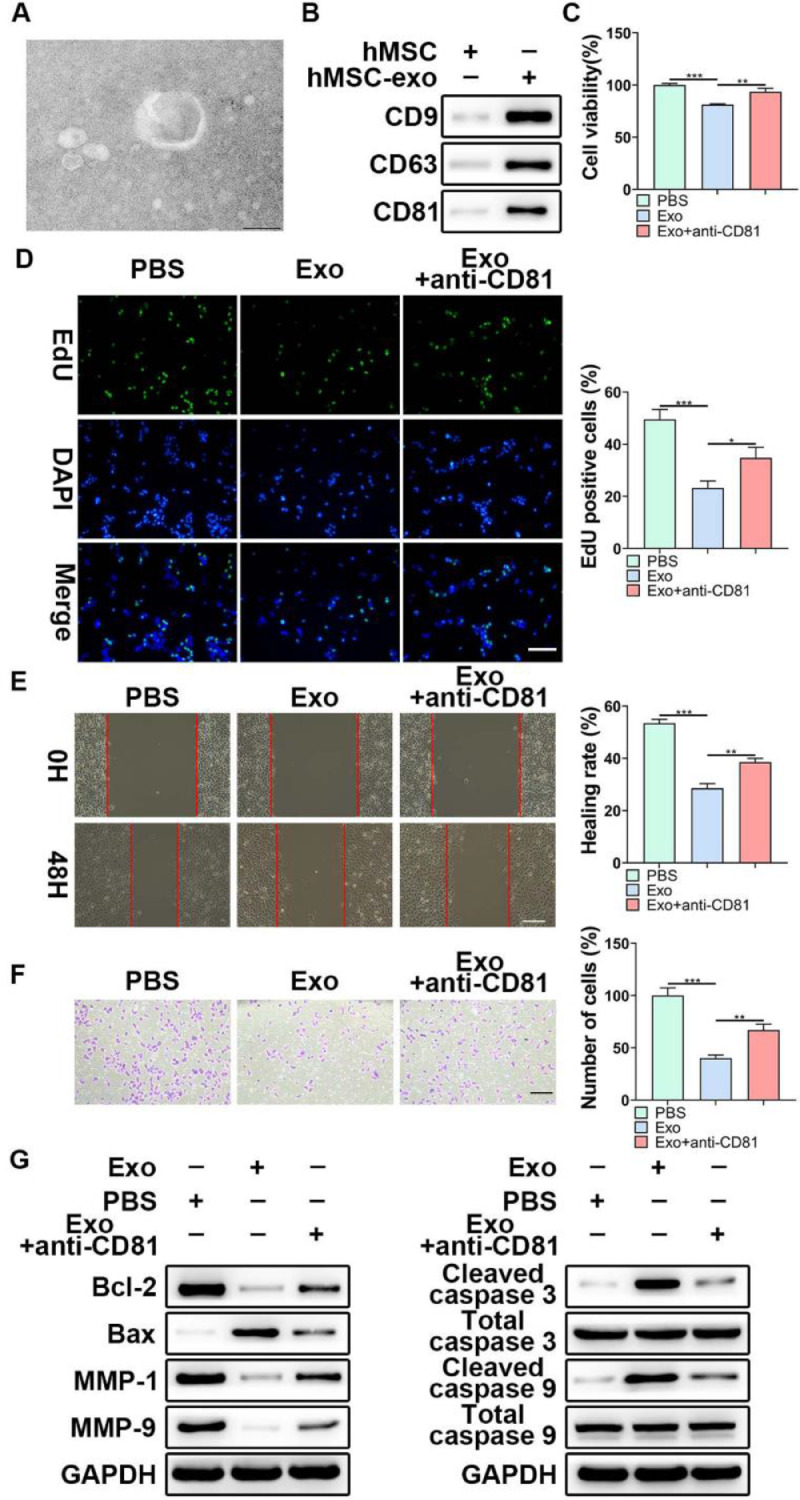
**The effects of MSCs-derived exosomes on LP development *in vitro*.** (A) TEM was applied to observe the shape of exosomes extracted from MSCs. (B) WB was utilized to detect the expression levels of exosomes-related indicators. (C‒F) LP cells were incubated with exosomes extracted from MSCs, followed by treatment with anti-CD81 neutralizing antibody, and then, subjected to the MTT (C), EdU (D), wound healing (E), and transwell (F) assays. (G) WB was employed to detect the expression levels of Bax, Bcl-2, MMP1, MMP9, caspase-3, cleaved-caspase-3, caspase-9, and cleaved-caspase-9 in LP cells, which were incubated with exosomes extracted from MSCs with or without anti-CD81 neutralizing antibody. *p < 0.05, ^⁎⁎^*p* < 0.01, ^⁎⁎⁎^*p* < 0.001.

Considering the functional role of miR-653-5p in LP cells, it was attempted to indicate whether exosomal miR-653-5p derived from MSCs could affect LP cell progression. First, exosomes were isolated from MSCs, and typically round or cup-shaped exosomal structures were observed under a transmission electron microscope (Fig. 2A). WB further validated the abundance of characteristic exosomal indicators (CD9, CD63, and CD81) in MSCs-derived exosomes (Fig. 2B). Subsequently, LP cells were incubated with Phosphate-Buffered Saline (PBS) or MSCs-secreted exosomes, followed by incubation with anti-CD81 antibody. It was revealed that MSCs-derived exosomes attenuated LP cell proliferation, which could be relieved via anti-CD81 treatment (Fig. 2C). Similarly, the results of the EdU assay illustrated that the striking reduction of EdU-incorporated LP cells caused by MSCs-secreted exosomes was abated by anti-CD81 treatment (Fig. 2D). Furthermore, the results indicated that exosomes from MSCs played a repressive function in the migration and invasion of LP cells, and anti-CD81 treatment led to the recovery of migration and invasion of LP cells (Fig. 2E, F). In accordance with the above-mentioned findings, MSCs-derived exosomes decreased the expression levels of Bcl-2, MMP-1, and MMP-9, while enhancing the expression levels of Bax, cleaved caspase-3, and cleaved caspase-9, and the effects of MSCs-derived exosomes on the expression levels of apoptosis-related proteins were abrogated when LP cells were subjected to anti-CD81 treatment (Fig. 2G). Collectively, MSCs-secreted exosomes hindered the malignant behaviors of LP cells.

### Exosomes mediated the delivery of exosomal miR-653-5p secreted by MSCs to LP cells

**Fig. 3:**
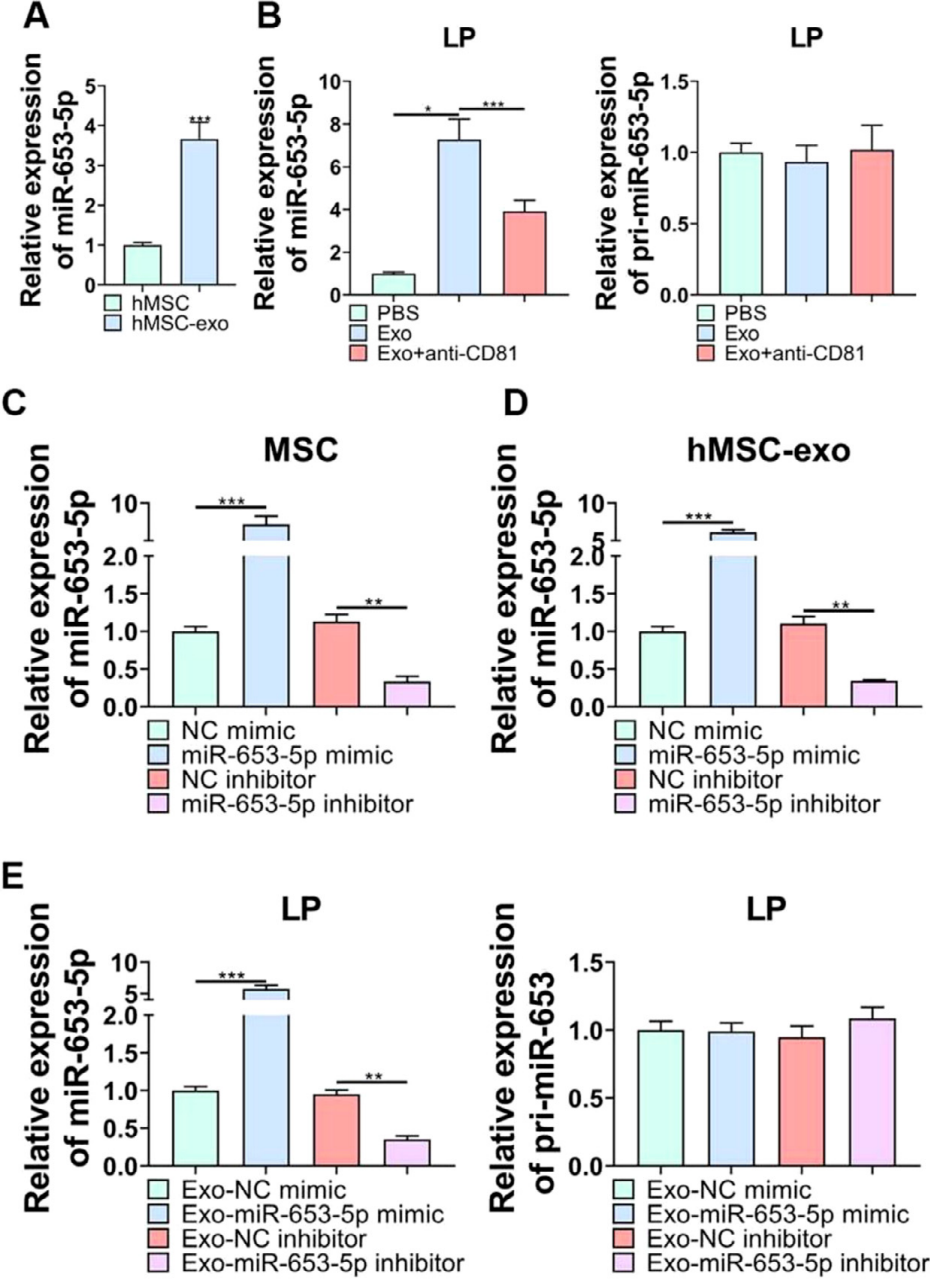
**The delivery of miR-653-5p to LP cells via MSCs-derived exosomes.** (A) qPCR was used for detection of miR-653-5p expression in MSCs and MSCs-derived exosomes. (B) LP cells were incubated with exosomes extracted from MSCs with or without anti-CD81 neutralizing antibody, and the expression levels of miR-653-5p and pri-miR-653 in LP cells were measured by qPCR. (C) The expression level of miR-653-5p was detected in MSCs transfected with miR-653-5p mimics, inhibitors and their negative controls. (D) qPCR analysis of the expression level of miR-653-5p in exosomes secreted by MSCs transfected with miR-653-5p mimics, inhibitors and their negative controls. (E) LP cells were cultured with exosomes from transfected MSCs, and the expression levels of miR-653-5p and pri-miR-653 in LP cells were determined by qPCR. *p < 0.05, ^⁎⁎^*p* < 0.01, ^⁎⁎⁎^*p* < 0.001.

According to qPCR analysis, miR-653-5p was found to be upregulated in MSCs-secreted exosomes as opposed to MSCs (Fig. 3A). Afterwards, it was revealed that the expression level of miR-653-5p in LP cells was elevated by MSCs-derived exosomes, and then, declined by administration of anti-CD81, while the expression level of pri-miR-653 was not significantly changed in LP cells following different treatments (Fig. 3B). Besides, the expression level of miR-653-5p was prominently augmented in MSCs-derived exosomes when miR-653-5p was overexpressed in MSCs, and the expression level of miR-653-5p was downregulated in exosomes extracted from miR-653-5p-silenced MSCs (Fig. 3C, D). In order to certify that miR-653-5p derived from BMSCs could be transferred to LP cells, it was attempted to co-culture LP cells with exosomes extracted from MSCs transfected with miR-653-5p mimics, miR-653-5p inhibitors or their negative controls. As shown in Fig. 3E, miR-653-5p was highly expressed in LP cells after co-cultured with exosomes from miR-653-5p-overexpressed MSCs, and the expression level of miR-653-5p was downregulated in LP cells via exosomes secreted by miR-653-5p-silenced MSCs. Meanwhile, no significant alteration was observed in pri-miR-653 following co-culture. Taken together, the results clarified that MSCs-derived exosomes could efficiently deliver miR-653-5p to LP cells.

### MiR-653-5p from MSCs-secreted exosomes suppressed proliferation, migration, and invasion of LP cells

**Fig. 4:**
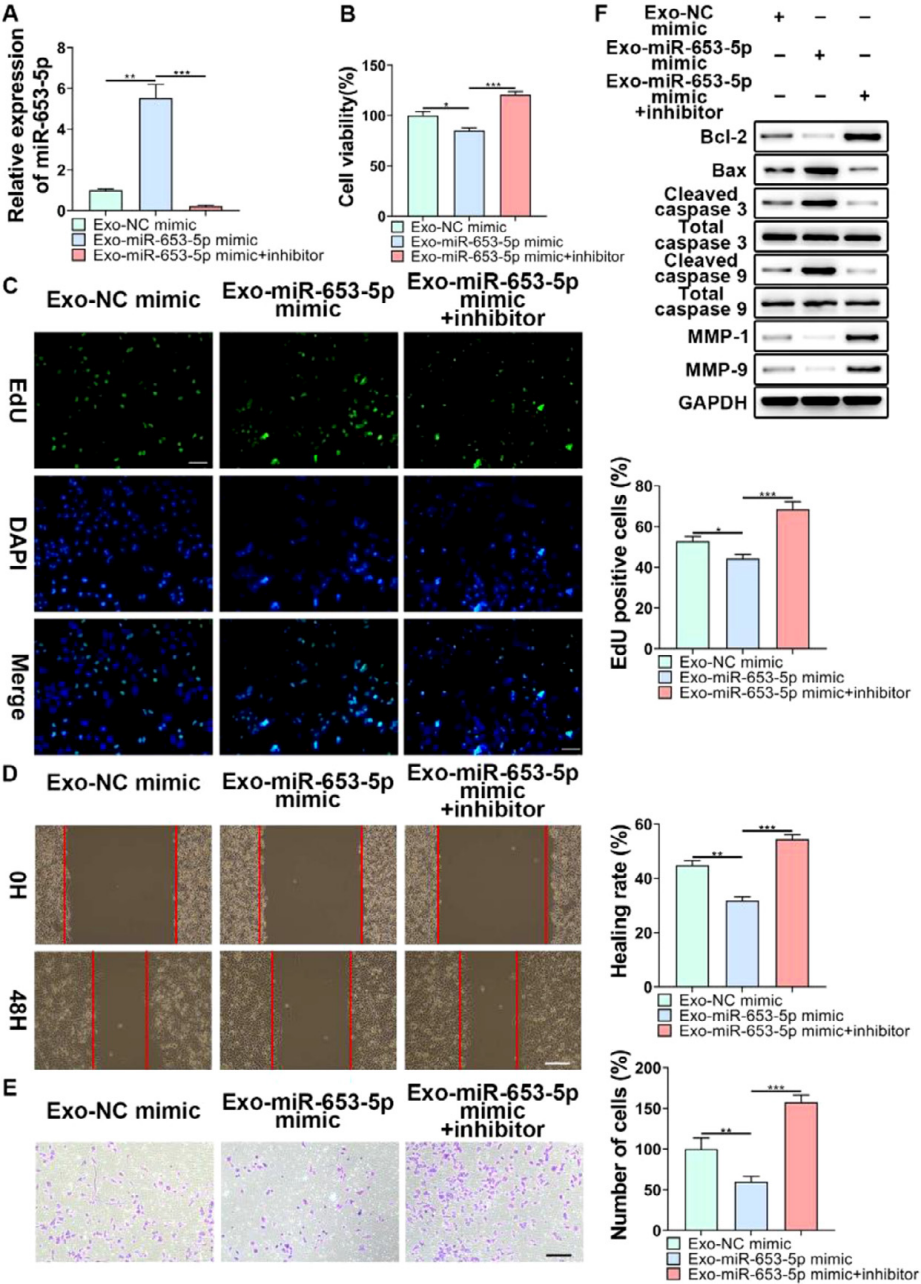
**The function of miR-653-5p from MSCs-derived exosomes in biological behaviors of LP cells.** (A) LP cells, transfected with miR-653-5p inhibitors, were incubated with exosomes isolated from miR-653-5p-upregulated MSCs, and qPCR was employed for the measurement of miR-653-5p expression in LP cells. (B-E) LP cells, transfected with miR-653-5p inhibitors, were incubated with exosomes isolated from miR-653-5p-upregulated MSCs, and then, subjected to MTT (B), EdU (C), wound healing (D), and transwell (E) assays. (F) LP cells, transfected with miR-653-5p inhibitors, were incubated with exosomes isolated from miR-653-5p-upregulated MSCs, and the expression levels of Bax, Bcl-2, MMP1, MMP9, caspase-3, cleaved-caspase-3, caspase-9, and cleaved-caspase-9 were detected by WB. *p < 0.05, ^⁎⁎^*p* < 0.01, ^⁎⁎⁎^*p* < 0.001.

To further validate the regulatory role of MSCs-secreted exosomes in LP cell progression by transferring miR-653-5p, the expression level of miR-653-5p in MSCs was upregulated, and then, the co-culture of miR-653-5p-silenced LP cells with MSCs-secreted exosomes isolated from transfected MSCs was carried out. It was verified by qPCR that the expression level of miR-653-5p in LP cells was elevated after treatment with exosomes derived from MSCs transfected with miR-653-5p, which could be restrained by miR-653-5p inhibitors (Fig. 4A). The results of the MTT assay demonstrated that exosomes derived from miR-653-5p-overexpressed MSCs inhibited the proliferation of LP cells, which could be rescued by silencing of miR-653-5p in LP cells (Fig. 4B). Consistently, the number of EdU-positive cells reduced by miR-653-5p-overexpressed exosomes from MSCs could be partly recovered owing to downregulation of miR-653-5p in LP cells (Fig. 4C). Wound healing and transwell assays illuminated that knockdown of miR-653-5p abolished the function of exosomes secreted by miR-653-5p-upregulated MSCs in migration and invasion of LP cells (Fig. 4D, E). In addition, overexpression of miR-653-5p in MSCs-secreted exosomes resulted in the decreased expression levels of Bcl-2, MMP-1, and MMP-9, as well as the upregulated expression levels of Bax, cleaved caspase-3, and cleaved caspase-9 in LP cells, which could be blocked by silencing of miR-653-5p (Fig. 4F). In summary, the above-mentioned findings indicated that MSCs-secreted exosomal miR-653-5p could exert an inhibitory influence on the aggressive phenotypes of LP cells.

### BZW2 acted as a downstream effector of miR-653-5p in LP cells

**Fig. 5:**
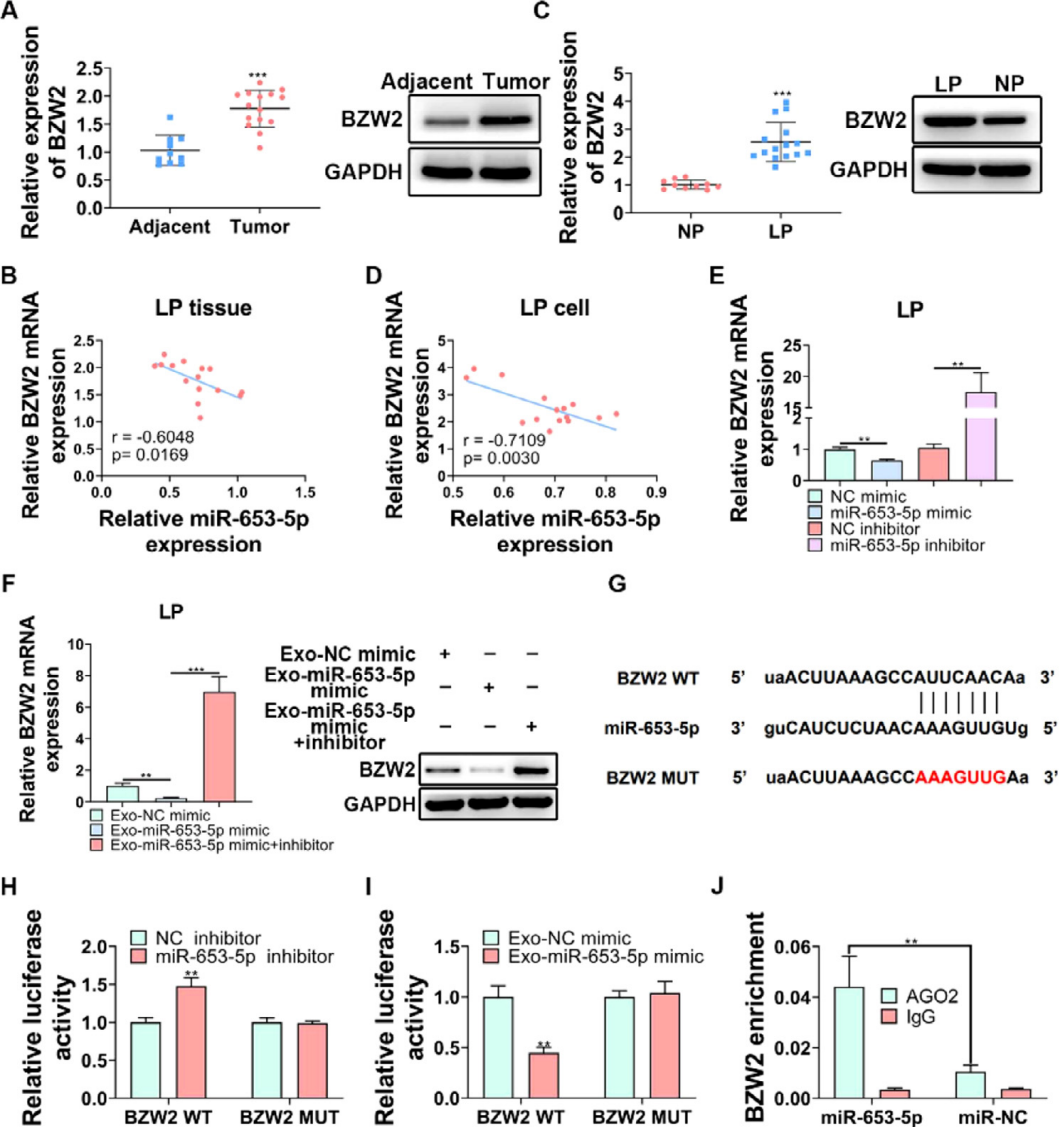
**BZW2 is a target gene of miR-653-5p in LP.** (A) The expression level of BZW2 was measured in LP tissues and adjacent normal tissues by WB and qPCR. (B) The correlation analysis of the expression levels of miR-653-5p and BZW2 in LP tissues. (C) The expression level of BZW2 in LP cells and normal pharyngeal epithelial cells was examined by WB and qPCR. (D) The association between the expression levels of miR-653-5p and BZW2 in LP cells was determined by the Spearman correlation analysis. (E) qPCR was utilized to detect the expression level of BZW2 in miR-653-5p-silenced or miR-653-5p-overexpressed LP cells. (F) LP cells, transfected with miR-653-5p inhibitors, were incubated with exosomes isolated from miR-653-5p-upregulated MSCs, and the expression level of BZW2 was measured by qPCR and WB. (G) The binding site between miR-653-5p and BZW2. (H‒J) The interaction of miR-653-5p and BZW2 was confirmed by dual-luciferase reporter (H, I) and RIP (J) assays. ^⁎⁎^*p* < 0.01, ^⁎⁎⁎^*p* < 0.001.

BZW2 has been proved to be involved in LP cell progression,^27^ therefore, it was attempted to assess its functional role in LP. The expression level of BZW2 was significantly higher in LP tissues than that in adjacent normal tissues (Fig. 5A, B). Importantly, the correlation analysis revealed a negative association between the expression levels of miR-653-5p and BZW2 in LP tissues (Fig. 5B). In addition, the expression level of BZW2 was upregulated and negatively correlated with the expression level of miR-653-5p in LP cells (Fig. 5C, D). To verify this correlation, LP cells were transfected with miR-653-5p mimics or miR-653-5p inhibitors for upregulation or silencing of miR-653-5p, and it was found that BZW2 was efficiently inhibited by miR-653-5p overexpression, while the expression level of BZW2 was enhanced by downregulation of miR-653-5p (Fig. 5E). Notably, exosomes secreted by miR-653-5p-upregulated MSCs suppressed the expression of BZW2, and restoration of the expression level of BZW2 when LP cells were treated with miR-653-5p inhibitors was observed (Fig. 5F). Moreover, there was a binding site between miR-653-5p and BZW2 (Fig. 5G). Dual-luciferase reporter assay demonstrated that the luciferase activity of BZW2-WT was only impaired by miR-653-5p inhibitors, while that of mutant isoform exhibited no change (Fig. 5H). Moreover, it was revealed that the upregulated expression of miR-653-5p in MSCs-secreted exosomes significantly decreased the luciferase activity of BZW2-WT, while it failed to affect the BZW2-MUT group (Fig. 5I). In addition, RIP assay clarified that exosomes derived from MSCs transfected with miR-653-5p mimic promoted effective enrichment of BZW2 in AGO2 antibody, rather than in IgG antibody (Fig. 5J). Collectively, these data confirmed the targeting relationship between miR-653-5p and BZW2 in LP.

### Exosomal miR-653-5p retarded the aggressive traits of LP cells through targeting BZW2

**Fig. 6:**
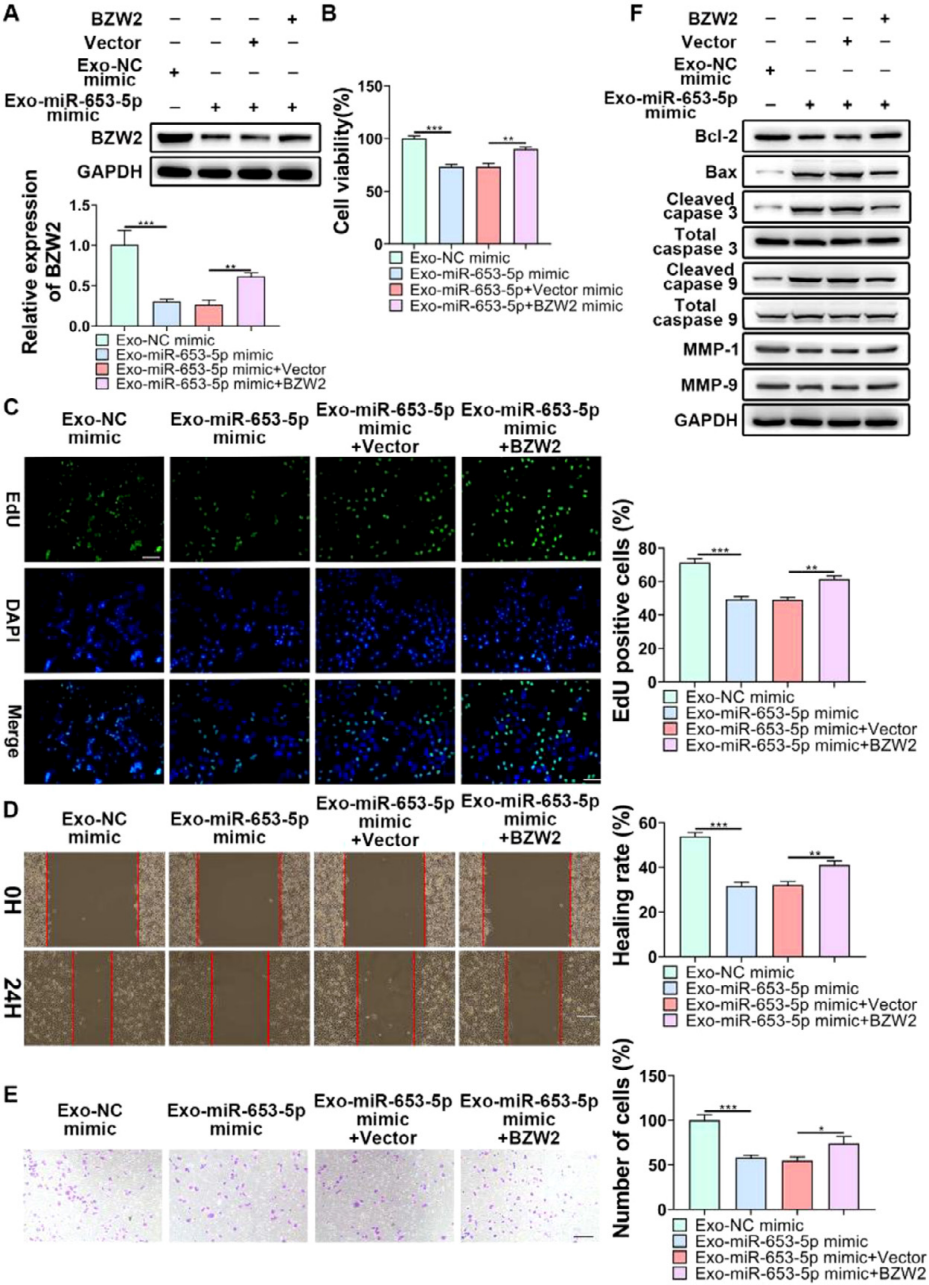
**Exosomal miR-653-5p inhibited the malignancy of LP in a BZW2-dependent manner.** (A) BZW2-overexpressed LP cells were incubated with exosomes derived from MSCs transfected with miR-653-5p mimics, and the expression level of BZW2 in LP cells was detected by qPCR and WB. (B-E) BZW2-overexpressed LP cells were incubated with exosomes derived from MSCs transfected with miR-653-5p mimics, and then, subjected to MTT (B), EdU (C), wound healing (D), and transwell (E) assays. (F) BZW2-overexpressed LP cells were incubated with exosomes derived from MSCs transfected with miR-653-5p mimics, and WB was employed to detect the expression levels of Bax, Bcl-2, MMP1, MMP9, caspase-3, cleaved-caspase-3, caspase-9, and cleaved-caspase-9. *p < 0.05, ^⁎⁎^*p* < 0.01, ^⁎⁎⁎^*p* < 0.001.

To indicate whether exosomal miR-653-5p could influence biological behaviors of LP cells in a BZW2-dependent manner, it was attempted to upregulate the expression level of miR-653-5p in MSCs and to overexpress BZW2 in LP cells, and then, co-incubation of MSCs-secreted exosomes and LP cells was performed. The results of qPCR and WB confirmed that exosomal miR-653-5p from transfected MSCs induced decreased expression of BZW2, which was restored by the enforced expression level of BZW2 (Fig. 6A). Furthermore, the results of MTT (Fig. 6B) and EdU (Fig. 6C) assays revealed that overexpressed miR-653-5p secreted by MSCs-derived exosomes resulted in the suppression of LP cell proliferation, which could be partly recovered by BZW2 overexpression in LP cells. As expected, migration and invasion of LP cells were hindered by exosomal miR-653-5p from MSCs-derived exosomes, and then, reversed by the upregulation of BZW2 in LP cells (Fig. 6D, E). Additionally, overexpression of BZW2 counteracted the role of MSCs-derived exosomal miR-653-5p in regulating the expression levels of apoptosis-relevant proteins (Fig. 6F). Based on these results, the authors concluded that MSCs-derived exosomal miR-653-5p played a suppressive role in LP development via targeting BZW2.

## Discussion

LP is a type of benign tumor with a high incidence in children, and it is prone to relapse. Despite the lower malignant conversion rate of LP, it remains a risk factor for respiratory disease.^28^, ^29^, ^30^ It is essential to explore the underlying mechanism of LP pathogenesis. To date, several miRNAs have been found to participate in the development of LP. For instance, Liu et al. indicated that miR-4500 overexpression weakened the proliferation of laryngeal papilloma cells via inhibiting the expression level of BZW2.^27^ Yin et al. demonstrated that miR-1224-5p alleviated HPV-induced laryngeal papilloma through suppression of 2-Oxoglutarate and Iron-Dependent Oxygenase Domain Containing 1 (OGFOD1).^31^ A growing body of evidence certified that miR-653-5p could serve as a repressive factor in diverse types of human cancer, such as neuroblastoma,^32^ breast cancer, non-small lung cancer,^33^ and melanoma.^34^ However, the modulatory role of miR-653-5p in LP has not been fully clarified. In the present study, the downregulated expression level of miR-653-5p in LP tissues and cells was measured. Functional assays demonstrated that miR-653-5p contributed to LP cell proliferation, migration, and invasion, while enhanced LP cell apoptosis. Consistent with previously reported findings, the inhibitory role of miR-653-5p in LP cell progression was confirmed.

Recently, exosomes, as a new hot spot, have noticeably attracted researchers' attention. Because exosomes contain bioactive substances that possess specific function, such as circRNAs, lncRNAs, and miRNAs,^35^ their biological role in various types of human cancer has been investigated. Importantly, increasing evidence has concentrated on the contribution of exosomal miRNAs to tumor progression. For instance, Kim et al. demonstrated that tumor-secreted exosomal miR-1260b facilitated the growth and metastasis of non-small cell lung cancer cells via negatively modulating Homeodomain-Interacting Protein Kinase 2 (HIPK2).^36^ Qin et al. pointed out that the inhibition of exosomal miR-34c derived from tumor cells accelerated cholangiocarcinoma progression through inducing cancer-related fibroblast activation.^26^ In addition, Liu et al. reported that exosomal miR-181a from human umbilical cord MSCs alleviated the malignant behaviors of nasopharyngeal carcinoma through targeting lysine-specific demethylase 5C (KDM5C).^37^ Based on the above-mentioned findings, the present study, it was attempted to indicate whether MSCs-derived exosomal miR-653-5p could affect the biological behaviors of LP cells. Thus, MSCs-derived exosomes were isolated, and the identification of exosomes was performed by TEM. Moreover, the results indicated that MSCs-derived exosomes played a suppressive role in LP development. More importantly, exosomes successfully delivered miR-653-5p to LP cells. Furthermore, after the co-incubation of MSCs-derived exosomes with LP cells, it was confirmed that exosomal miR-653-5p secreted by MSCs suppressed the proliferation, migration, invasion, and apoptosis of LP cells.

BZW2 participates in the development of multiple types of cancer, such as hepatocellular carcinoma,^38^ colorectal cancer,^39^ and bladder cancer.^40^ Notably, Liu et al. demonstrated that upregulation of BZW2 by LINC00174 facilitated the malignant behaviors of laryngeal papilloma cells.^27^ It was validated that BZW2 accelerated the development of hepatocellular carcinoma via the c-Myc pathway.^38^ Silencing of BZW2 retarded cell growth in osteosarcoma through regulating the Akt/mTOR signaling pathway.^41^ In accordance with a previous study, BZW2 was highly expressed in LP cells and tissues. Regarding the negative correlation between BZW2 and miR-653-5p confirmed by the Spearman correlation test, BZW2 was selected for an in-depth study. Subsequently, BZW2 was nominated as the target of miR-653-5p based on the dual-luciferase reporter and RIP assays. More importantly, exosomal miR-653-5p negatively regulated the expression level of BZW2 in LP cells and acted as an inhibitor in malignant behaviors of LP cells in a BZW2-dependent manner.

## Conclusions

In summary, the downregulation of miR-653-5p in LP cells and tissues was revealed, and the inhibitory function of miR-653-5p in the malignant features of LP cells was validated. Furthermore, it was confirmed that MSCs-derived exosomal miR-653-5p played an inhibitory role in LP progression through suppressing the expression level of BZW2, providing a new idea for the application of MSCs-derived exosomes in LP therapy.

## Ethics approval and consent to participate

This study was performed in accordance with the Declaration of Helsinki, as well as national and international guidelines. This study was approved by the Ethics Committee of Hunan Children's Hospital.

## Funding

This research received no specific grant from any funding agency in the public, commercial, or not-for-profit sectors.

## CRediT authorship contribution statement

**Binya Hu:** Visualization, Investigation, Writing – original draft. **Min Huang:** Investigation. **Lihua Tao:** Investigation. **Yun Li:** Investigation. **Yuting Kuang:** Investigation. **Guangliang Liu:** Investigation. **Sijun Zhao:** Visualization, Investigation, Writing – review & editing.

## Conflicts of interest

The authors declare no conflicts of interest.

## Appendix Supplementary materials

Image, application 1
