# Supplementary material for: Mesenchymal stem cells-derived exosomal miR-653-5p suppresses laryngeal papilloma progression by inhibiting BZW2
Source: Clinics (Sao Paulo). 2022 Dec 5;78:100129. doi: 10.1016/j.clinsp.2022.100129 (PMC9723928; doi:10.1016/j.clinsp.2022.100129)
Supplement: Supplementary file 1 [file mmc1.docx]

**CLINICS-D-22-00140 – Supplementary Material**

**Supplementary Table 1** Sequences of PCR primers used in this study.

| miR-653-5p | Forward (5’-3’) | TTGAAACATTCTCTACTGAAC |
| --- | --- | --- |
|  | Reverse (5’-3’) | GAACATGTCTGCGTATCTC |
| GAPDH | Forward (5’-3’) | TGTGGGCATCAATGGATTTGG |
|  | Reverse (5’-3’) | ACACCATGTATTCCGGGTCAAT |
| BZW2 | Forward (5’-3’) | AACAGGCCAGCGGTTCAAAA |
|  | Reverse (5’-3’) | CCCCTGGACAAGTGTATCCCT |
